# Effect of Inorganic Dietary Selenium Supplementation on Selenoprotein and Lipid Metabolism Gene Expression Patterns in Liver and Loin Muscle of Growing Lambs

**DOI:** 10.1007/s12011-015-0592-0

**Published:** 2015-12-23

**Authors:** Edyta Juszczuk-Kubiak, Kamila Bujko, Monika Cymer, Krystyna Wicińska, Mirosław Gabryszuk, Mariusz Pierzchała

**Affiliations:** Laboratory of Genome and Transcriptome Sequencing, Department of Molecular Biology, Institute of Genetics and Animal Breeding, Polish Academy of Sciences, Jastrzębiec, Poland; Department of Animal Breeding, Institute of Genetics and Animal Breeding, Polish Academy of Sciences, Jastrzębiec, Poland; Department of Genomics, Institute of Genetics and Animal Breeding, Polish Academy of Sciences, Jastrzębiec, Poland

**Keywords:** Selenium supplementation, Lambs, mRNA expression, Selenoprotein genes, Lipid metabolism genes, Liver, Muscle tissue

## Abstract

Effect of selenium (Se) supplementation on the selenoprotein and lipid metabolism gene expression patterns in ruminants, especially in lambs is not yet fully understood. The aim of study was to evaluate the effect of Se supplementation on the messenger RNA (mRNA) expression patterns of selected selenoproteins and genes related to lipid metabolism in growing lambs. The experiment was conducted on 48 Polish Merino lambs divided into two groups (*n* = 24): control (C)—lambs fed with a basal diet (BD) with no Se supplementation, and supplemented (S)—lambs fed with a BD, supplemented with 0.5 mg Se/kg as sodium selenate for 8 weeks. Expression of 12 selenoproteins and six genes related to lipid metabolism was analyzed in the liver and *longissimus dorsi* (LD) muscle of growing lambs by qPCR. Significant differences were found in the expression of *GPX1*, *GPX2*, *SEPM*, *SEPW1*, *SEP15*, *SEPGS2,* and *TXNRD1* in the liver, and *GPX1*, *SEPP1*, *SEPN1*, *SEPW1*, *SEP15,* and *MSRB1* in the LD muscle between S and C lambs. Se supplementation mainly upregulated *SEPW1*, *SEP15* (*P* < 0.001; *P* < 0.01) mRNA expression in the liver, and *GPX1*, *SEPP1*, *SEPN1*, *SEPW1* (*P* < 0.001; *P* < 0.01) in the muscle of S group. On the other hand, significant decrease in *GPX2* (*P* < 0.01), *SEPM* (*P* < 0.001), and *SEPHS2* (*P* < 0.01) mRNA expression levels were observed in the liver of S group of lambs. Se supplementation did not affect *PON1*, *LXRα,* and *PPARα* mRNA expression levels, but a significant increase in mRNA levels of *APOE* and *LPL* in the LD muscle (*P* < 0.05) as well as *LPL* (*P* < 0.05) in the liver were noticed in the group of Se supplemented lambs. Our study confirmed that, in lambs, similarly to other species, mRNA expression patterns of several selenoproteins highly depend on dietary Se levels, and their expression is ruled by hierarchical principles and tissue-specific mechanisms. Moreover, the study showed that changes Se intake leads to different levels of genes expression related with lipid metabolism.

## Introduction

Along with the development of nutrigenomics, increased interest in the molecular mechanism of macro- and micronutrient action, its influence on human and animal genomes, and consequently—their health. Particular attention was aimed at selenium (Se), a trace mineral and an essential bioactive nutrient in the human and animals’ diet [1]. It was indicated that Se plays an important role in the nervous system biology, immune functions, reproduction, muscle metabolism, chemoprevention, redox reactions, and many others [2].

In livestock animals, including ruminants, Se deficiency induces a number of diseases, like nutritional muscular dystrophy [3], cardiomyopathy [4], hepatic degeneration [5], and reproduction disturbances [6].

In recent years, some studies showed that physiological functions of Se are considered to be mediated through selenoproteins [7]. At least 25 selenoprotein genes in animals and humans have been identified and several families of selenoproteins have been classified according to their biological function: (1) peroxidase and reductase activity; (2) hormone metabolism; (3) protein folding; (4) redox signalling; (5) Sec synthesis, and (6) selenium transport [2, 6, 8, 9]. Among these selenoproteins, the important are glutathione peroxidase family (GPXs), thioredoxin reductase (TRXRs), iodothyronine deiodinase (DIOs), and selenoprotein W, N, and P [2, 10]. In recent years, new groups of selenoproteins have been classified including SEP15, SEPHS2, SEPM, MSRB1, and selenoproteins I, N, V, O, H, T, K, and S, but the function of many of them is still unknown [8].

Selenoproteins are extensively expressed in animals, but the expression patterns and the effect of Se on their messenger RNA (mRNA) levels varied from different tissues and species. Different levels of expression were observed in the brain, liver, spleen, testis, thyroid, as well as in cardiac and skeletal muscles [11]. Selenoproteins, such as *GPX*s, were found to express in the liver, kidney, and muscle [5], whereas *SELW* and *SELN mRNA* transcripts were detected in various kinds of tissues, especially more abundant in muscles [12].

The effect of dietary Se deficiency and Se supplementation on the expression of selenoprotein genes has been studied in rats [13], mice [14], pigs [3, 15], cattle [16], and chickens [5]. The synthesis of selenoproteins is affected by the levels of Se supplementation, and their mRNA expression is controlled in a hierarchical manner [17]. In mammals *GPX1*, *GPX3*, and *SEPP1* mRNA levels were dramatically decreased with Se deficiency [18], in contrast to several other selenoproteins including *GPX4*, *SEPP1*, *TXNRD1*, and *DIO1*, which mRNA levels were relatively unchanged in the liver, kidney, thyroid, and other tissues [15, 19].

Recently, studies on animal models [20] and well as human trial [21] showed that changes in Se intake lead to altered mRNA expression of genes associated with the cholesterol and lipid metabolism [22]. Se deficiency was related to an increase in the plasma cholesterol, whereas Se supplementation led to decrease in total cholesterol and triglyceride levels [23]. Pinto et al. [24] reported that high-Se diets increased gene expression of *FoxO1* and *PGC*-*1*α in the porcine skeletal muscle and enhanced mRNA expression of *SREPP*-*1* and *LPL* in the adipose tissue. A number of studies also confirmed that Se supplementation can affect lipid metabolism in ruminants [25]. The effect of Se supplementation on the modification of fatty acid composition in the direction of increasing the content of PUFAs, especially CLA isomers in lambs’ meat and liver [26] as well as in cow’s milk [27] and goat [28] has been reported.

Currently, the regulation of specific gene expression in response to changes in Se nutrition is being deeply studied in rodents and pigs as well as in humans, but there are no studies in ruminants, especially in lambs. Our objective in this study, therefore, was to investigate how oral Se supplementation in form of selenate (0.5 mg Se/kg) change the mRNA expression patterns of selected selenoprotein genes (*GPX1*, *GPX2*, *GPX4*, *SEPP1*, *SEPM*, *SEPN1*, *SEPW1*, *SELO*, *SEP15*, *SEPHS2*, *TXNRD1*, and *MSRB1*) and genes related to lipid metabolism (*LPL*, *LXRα*, and *PPARα*) as well as cholesterol metabolism (*PON1*, *APOE*, and *DHCR24*) in the liver and loin (*longissimus dorsi*) muscle of growing Polish Merino lambs.

## Materials and Methods

### Animals and Diet

The experiment was conducted on 48 single born Polish Merino ram-lambs housed in the Institute Farm of the IGAB PAS in Jastrzębiec, Poland. At 8 weeks all lambs were weaned and fed with a basal diet (BD) ad libitum for 2 weeks to adjust their Se status (Table 1). After adaptation period, the animals were transferred individually to straw-bedded pens and divided into two groups (*n* = 24): I—control (C)—lambs fed ad libitum with a basal diet (BD) with no Se supplementation, and II—supplemented (S)—lambs fed ad libitum with a BD supplemented *per os* with 0.5 mg Se/kg as sodium selenate (Na_2_SeO_4_) for 8 weeks. The BD was composed of oats (40 %), triticale (29 %), soybean meal (19.5 %), rapeseed oilcake (10 %), limestone (1 %), and NaCl (0.5 %). Concentration of native Se in BD was 0.1 mg/kg dry matter (DM). Additionally, during the supplementation period about 0.1 kg meadow hay/lamb/day was offered in order to ensure proper rumen function (0.09 mg Se/kg DM). The individual intake of BD was recorded daily. Body weights of individual lambs were recorded at the beginning of Se supplementation and then weekly. To prepare plasma samples, blood was collected from individual lambs at the beginning and final of the experiment. The plasma Se concentration was determined by inductively coupled plasma atomic emission spectroscopy (ICP-AES) (Optima 5300 DV, Perkin Elmer). At 18 weeks, lambs were slaughtered and liver and *longissimus dorsi* (LD) samples were collected. The tissue samples were immediately frozen by liquid nitrogen and stored at −80 °C.

**Table 1:** Chemical composition of feeds (g/kg DM)

Item	Basal diet (BD)^a^	Meadow hay
Organic matter	912	922
Crude protein	192	82
Crude fiber	106	303
Ether extract	36	20
NDF	282	566
ADF	124	361
Se (selenate) (mg/kg DM)	0.1	0.08
Zn (mg/kg DM)	27.2	15.1

^a^The concentrate was composed of 40 % oat meal, 29 % wheat-rye meal, 19.5 % soybean meal, 10 % rapeseed cake, 1 % limestone, and 0.5 % NaCl

### RNA Isolation and cDNA Synthesis

Total RNA was isolated with the High Pure RNA Tissue Kit (Roche Diagnostics, Switzerland) from liver and *longissimus dorsi* muscle samples, according to the manufacturer’s instructions. A qualitative and quantitative assessment of the isolated RNA was performed on ND-1000 NanoDrop (NanoDrop Technologies, USA) spectrophotometer. Only samples with more than 100 ng RNA and absorbance ratios A260/280 and A260/230 of around 2.0 were used for further analyses. To confirm the RNA quality, a quick electrophoretic analysis was conducted using a BioAnalyzer (Agilent, USA). The RNA was reverse transcribed into cDNA with the Transcription First Strand cDNA Synthesis Kit (Diagnostics, Switzerland). 1.0 μg of RNA was denatured at 65 °C for 10 min in the presence of 50 μM oligo(dT). The reverse-transcription mixture (20 μl in total volume) contained 13 μl of RNA, 4 μl of reverse transcriptase buffer, 2 μl of 10 mM dNTP, 0.5 μl of protector RNase Inhibitor (40U/μl), and 0.5 μl of reverse transcriptase (20U/μl). The mixture was incubated in 50 °C for 60 min, then at 85 °C for 5 min, and finally stored at −20 °C.

### Primer Design

Primers for gene expression were designed with Primer3Plus (http://www.bioinformatics.nl/) in accordance to GenBank *Ovis aries* sequences and amplicon sequence complied with exon-exon boundaries (Table 2). The relative mRNA abundances of 17 genes were assayed. Selected targets included 12 selenoprotein genes (glutathione peroxidase 1, *GPX1*; glutathione peroxidase 2, *GPX2*; glutathione peroxidase 4, *GPX4*; selenoprotein 1, *SEPP1*; selenoprotein W1, *SEPW1*, selenoprotein N1, *SEPN1*; thioredoxin reductase 1, *TXNRD1*; selenoprotein *15*, *SEP15*; selenophosphate synthetase 2, *SEPHS2*; selenoprotein M, *SEPM*; selenoprotein O, *SELO*; methionine sulfoxide reductase B1, *MSRB1*), three genes associated with lipid metabolism (lipoprotein lipase, *LPL*; liver X receptor α, *LXRα*; peroxisome proliferator-activated receptor *α*, *PPARα*) and three genes involved in cholesterol metabolism (paraoxonase 1, *PON1*; apolipoprotein *E*, *APOE*; 24-dehydrocholesterol reductase, *DHCR24*).

**Table 2:** Genes and primers used for relative quantification by real-time PCR (qPCR) in the liver and *longissimus dorsi* muscle of lambs

Target gene	Primer sequences	Product length	GeneBank ID
*GAPDH*	F: ACCACTTTGGCATCGTGGAG	75 bp	NM_001190390.1
	R: GGGCCATCCACAGTCTTCTG		
*ACTB*	F: CTCTTCCAGCCTTCCTTCCT	178 bp	NM_001009784.1
	R: GCAGAAAGAGATCACTGCCC		
*GPX1*	F: CCTGGTCGTACTCGGCTTC	154 bp	XM_004018462.1
	R: CCTTCTCGCCATTCACCTC		
*GPX2*	F: GGGCAGTGCTGATTGAGAAT	273 bp	XM_004010720.1
	R: TTCAGGTAGGCGAAGACAGG		
*GPX4*	F: GGGAGTAATGCGGAGATCAA	210 bp	XM_004023249.1
	R: CATACCGCTTCACCACACAG		
*SEPP1*	F: GACCGTGGTTGCTCTTCTTC	222 bp	XM_004017013.1
	R: GGTTGGTCTTCTTCTTGTTGG		
*SEPM*	F: TGTCGCTGAGTGGAATTCG	210 bp	XM_012112767.1
	R: ACTGGAACCGTCTACAAGGC		
*SEPN1*	F: GGATCCTGGACCACAAGATG	217 bp	XM_004023208.1
	R: TGGCCGCAGGATATAGTAGC		
*SEPN1*	F: TCGTCGTCCGAGTTGTTTACT	210 bp	XM_004023205.1
	R: GAAACTTGCTCTCCGTGTCC		
*SELO*	F: AGGTTCGGATCCTTCGAGAT	164 bp	XM_004007676.1
	R: TGTCTGCACATGGTCTCC		
*SEPHS2*	F: TTCAACATGGCTACCCTCAA	167 bp	XM_004020905.1
	R: GCAATGATGGGCAGATTATG		
*SEP15*	F: TTCAAACGGTCTCTGCATTG	251 bp	XM_004002164.1
	R: TCCTGACGAAAGCTTGGACT		
*TXNRD1*	F: GCTTTGGAATGTGCTGGATT	193 bp	XM_004006684.1
	R: TCTCCTTCGATGGTTTGGTC		
*MSRB1*	F: TGTCGTTCTGCAGCTTCTTC	162 bp	XM_004020710.1
	R: CACTGTCAGCATGGATGGTC		
*PON1*	F: TACAGCCCAGATGACGTTCG	210 bp	XM_004007735.1
	R: TGTCACAGGGTCCACGGATA		
*LXRα*	F: GGTACAACCCTGGAAGTGAGA	160 bp	XM_004016449.1
	R: CAAGGCAAACTCAGCATCATT		
*DHCR24*	F: GCGGGTTGGAAAGTACAAGA	229 bp	XM_004002011.1
	R: ATGGGATGAAGACTCGATGC		
*APOE*	F: CTACGGCGAGACCTTCAACA	210 bp	XM_004016047.1
	R: CCCAAAGGGACGGGTATCTC		
*PPARα*	F: ATGGCTTCATAACCCGTGAG	206 bp	XM_004007050.1
	R: AATCCCCTCCTGCATTTTCT		
*LPL*	F: TTCAACCACAGCAGCAAAAC	210 bp	NM_001009394.1
	R: AAACTTGGCCACATCCTGTC		

### Quantitative Real-Time PCR Assays (qPCR)

qPCR was performed using LightCycler 96 System (Roche Diagnostics, Switzerland) in 96-well optical reaction plates with a total reaction volume of 10 μl. The reaction mixture consisted of 1 μl of each primer (20 pM, Table 2), 5 μl of SYBR Green Master Mix (Roche Diagnostics, Switzerland), 2 μl of PCR-grade water, and 50 ng of cDNA. The amplification program consisted of: 10 min of initial denaturation at 95 °C, 40 cycles of denaturation (10 s, 95 °C), annealing (9 s, 59–61 °C), and elongation (20 s, 72 °C). qPCR amplifications were conducted in triplicates for both target and reference genes. Negative controls containing the template RNA and all PCR reagents, but not reverse transcriptase, were included to determine the RNA purity from DNA contamination. The qPCR data were normalized using two reference genes: β-actin (*ACTB*) and glyceraldehyde 3-phosphate dehydrogenase gene (*GAPDH*). The relative mRNA abundance of target genes in tissue samples were determined using Δ cycle threshold (^Δ^Ct) method in which, the ^Δ^Ct value is the difference between the target and reference gene (^Δ^Ct = Ct^target^–Ct^reference^). For each of the target gene the normalized relative expression was calculated (2^−ΔΔCt^, were ^ΔΔ^Ct = ^Δ^Ct sample–^Δ^Ct control). The expression of the target gene was set as 1, assuming its Ct at 27 (selenoproteins), 30 (lipid metabolism genes) and the reference gene *GAPDH*/*ACTB* at 22 and its ^Δ^Ct was used as the control. PCR reactions of each sample were conducted in triplicate.

### Statistical Analysis

Fold-changes of mRNA expression between groups and its statistical significance were evaluated with ANOVA procedure (SAS 9.3, SAS Institute, Cary NC, USA). Data was presented as means ± SE and the significance level was set at *P* < *0*.05.

## Results

### Growth Performance and Se Concentration in Plasma

The average body weight of lambs at the start and after supplementation period was 14.2 ± 0.3 and 31.2 ± 0.5 kg, and there was no difference between C and S groups of lambs (*P* > 0.05). The average daily feed intakes were 0.97 ± 0.2 kg of BD and 0.085 kg of meadow hay per lamb and were similar among the C and S groups of lambs. There was no observed difference in consumption during the study (*P* > 0.05). Mean daily Se intake per lamb was 0.15 mg Se/kg for C group and 0.65 mg Se/kg for S group. Plasma Se concentrations before the experiment were 1.11 ± 0.09 μmol/l in C group and 1.12 ± 0.11 μmol/l in S group, respectively. At the end of experiment, the final plasma Se concentrations noticeably increased (*P* < 0.05) in lambs from S group (1.47 ± 0.19 μmol/l) vs. C group (1.19 ± 0.15 μmol/l) (*P* < 0.05).

### Abundance of Selenoproteins mRNA

**Fig. 1:**
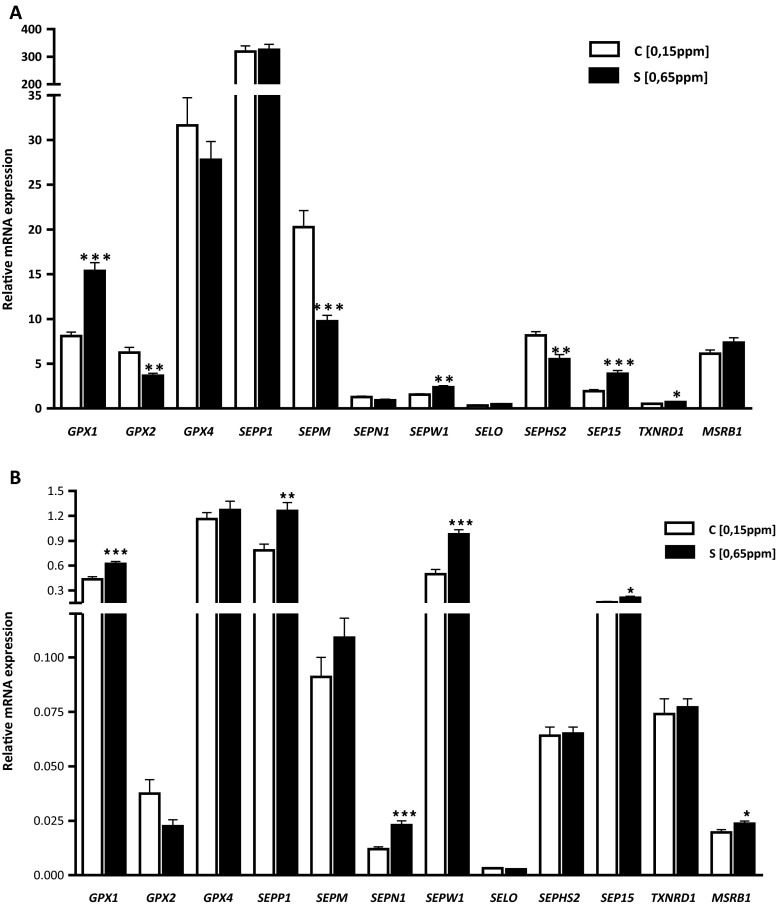
Effects of dietary Se concentrations on mRNA expression levels of selected selenoproteins (**a**) in the liver and (**b**) in the *longissimus dorsi* muscle. Data are mean ± SE, *n* = 24. *P < 0.05; **P < 0.01; ***P < 0.001

To investigate the effect of differences in Se status on mRNA expression of antioxidant selenoproteins, we determined the hepatic and muscle expression patterns of *GPX1*, *GPX2*, *GPX4*, *SEPP1*, *SEPM*, *SEPN1*, *SEPW1*, *SELO*, *SEP15*, *SEPHS2*, *TXNRD1*, and *MSRB1* genes. Our results showed that expression of these genes responded to dietary Se concentration in three patterns. In the first one, Se supplementation resulted in increased (*P* < 0.05, *P* < 0.01, *P* < 0.001) *GPX1*, *SEPW1*, *SEP15*, and *TXNRD1* mRNA levels in the liver (Fig. 1a) and *GPX1*, *SEPP1*, *SEPN1*, *SEPW1*, *SEP15*, and *MSRB1* in the LD muscle of S group (0.65 mg Se/kg) (Fig. 1b) compared with the C (0.15 mg Se/kg) group of lambs. In the second pattern, higher (*P* < 0.01, *P* < 0.001, *P* < 0.01) mRNA levels were manifested in the C group (0.15 mg Se/kg) than in the S group (0.65 mg Se/kg) including *GPX2*, *SEPM*, and *SEPHS2* in the liver; no significant differences were found in the LD muscle. The third pattern exhibited similar mRNA expression in the liver and muscle of S group; the expression of *GPX1*, *SEPW1*, and *SEP15* was upregulated by Se supplementation in both tissues. However, Se supplementation had nonsignificant effect on the expression of *GPX4*, *SEPP1*, *SEPN1*, *SELO*, and *MSRB1* in the liver, and *GPX2*, *GPX4*, *SEPM*, *SELO*, *SEPHS2*, and *TXNRD1* in the LD muscle.

### mRNA Levels of Lipid Metabolism-Related Genes

**Fig. 2:**
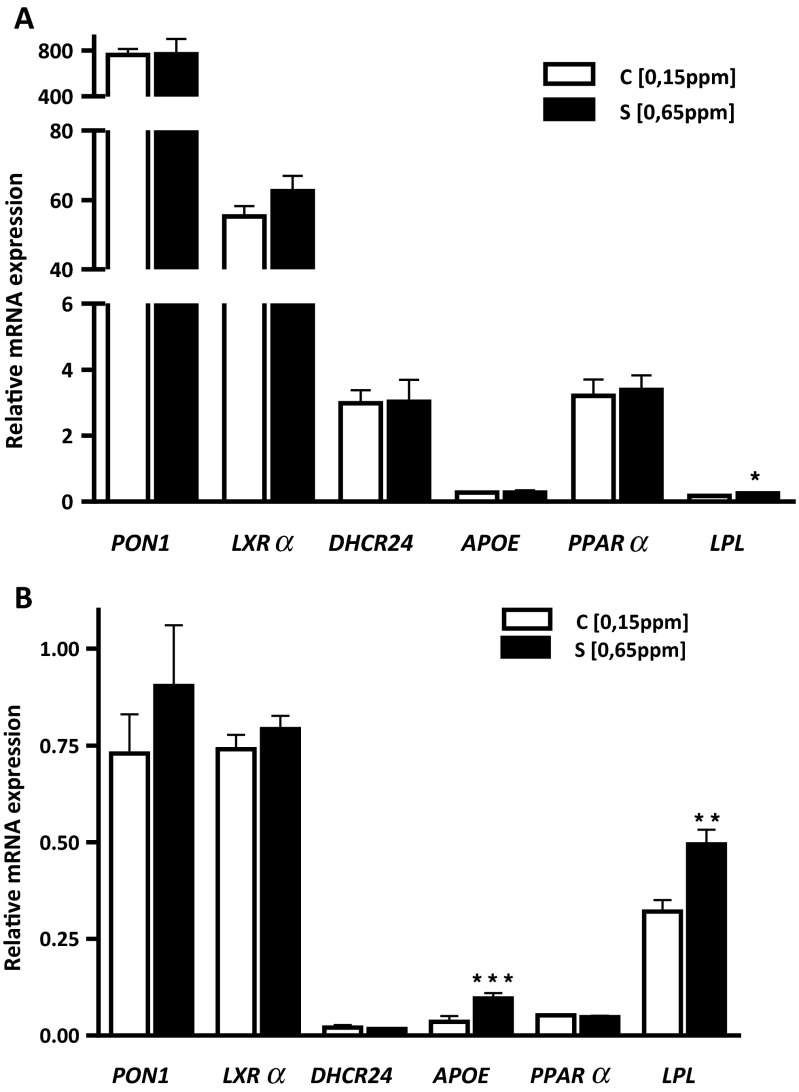
Effects of dietary Se concentrations on mRNA expression levels of lipid metabolism genes (**a**) in the liver and (**b**) in the *longissimus dorsi* muscle. Data are mean ± SE, *n* = 24. *P < 0.05; **P < 0.01

To investigate the effect of Se supplementation on mRNA level of genes involved in the regulation of the lipid and cholesterol metabolism, we assessed the hepatic and muscle expression patterns of *PON1*, *LXRα*, *LPL*, *APOE*, *PPARα*, and *DHCR24* genes. As shown in Fig. 2a, b, Se supplementation did not alter the mRNA levels of *PON1*, *LXRα*, and *PPARα*; mRNA levels of these genes remained unchanged both in the liver and LD muscle. On the other hand, Se supplementation significantly stimulated the mRNA expression of the *LPL* in both tissues and the *APOE* in the skeletal muscle. The significantly higher *LPL* mRNA level in the liver (*P* < 0.05) and LD muscle (*P* < 0.01) and *APOE* in the LD muscle (*P* < 0.001) was observed in the S (0.65 mg Se/kg) vs. C (0.15 mg Se/kg) group of lambs.

## Discussion

### General Remarks

In our study, the dietary Se concentration of the S lambs group was the upper recommended level by the NRC [29], whereas C group received 0.15 mg/kg DM of naturally occurring Se in feeds used. Body weight of lambs, daily feed intake, and gain efficiency in our experiment were not altered by the dietary Se status, which confirmed previous results in studies with lambs [30, 31], pigs [15], chickens [5], cattle [25], and goats [32]. On the other hand, the data of this study showed noticeably increased (*P* < 0.05) level of plasma Se concentration in lambs from S group vs. C group, which was consistent with findings reported in other species including pig [3, 15], chicken [18], goat [32], cattle [25], and turkey [33].

### Effect of Se Supplementation on mRNA Level of Selenoprotein Genes in the Liver and Muscle

qPCR analysis showed that investigated genes were expressed in both tissues, but relative higher mRNA levels were found in the liver, which indicates that the liver is more responsive to changes in dietary Se levels than the muscle. It is known, that the liver ranks higher than the muscles in the hierarchy of Se source and metabolism [34] and that the hepatic antioxidant system plays an important role in mammals [35]. As in the previous studies [30, 31], our results confirmed that changes in Se intake alter the mRNA level of lamb selenoproteins, and the effects varies significantly between different selenoproteins and different tissues. These variations could reflect the hierarchy of selenoproteins expression, indicating, that some are more sensitive to changes in Se intake than others [19].

Numerous studies have shown that *GPX1*, *SEP15*, and *SELW1* mRNA levels increased in response to dietary Se supplementation in the pig [15], chicken, [5] and sheep [30]. It was also demonstrated that hepatic expression of *GPX1*, *SEPW1*, and *SEP15* was highly and significantly downregulated by Se deficiency [18]. On the other hand, Liu et al. [11] reported that high-Se diet (3.0 mg Se/kg) decreased *SEPW1* mRNA level in the liver of pigs, compared with the 0.3 mg Se/kg diet. Our results showed that *GPX1*, *SEPW1*, *SEP15* transcripts were upregulated both in the liver and muscle of Se supplemented group of lambs (0.65 mg Se/kg). Increased mRNA levels of *GPX1* in the liver and muscle, *SEP15* in the liver, and *SEPW1* in the muscle were noticed. Moreover, muscle *GPX1* and *SEPW1* mRNA expression levels were more sensitive to regulation by Se status than *SEP15*. In the light of these results, one could hypothesize that *SEPW1* may participate in cell redox defense in mature muscle fibers and be involved in the prevention of skeletal muscle degeneration [3]. *SEPW1* is probably involved in the etiology of sheep nutritional myopathy, the white muscle disease [36]. Less is known about role of *SEP15*, but its antioxidative function was recently indicated in some mammalian and human cell lines [37]. Our results showed that Se supplementation had a significant effect on *GPX1* and *SEPW1* mRNA expression both in the liver and muscle, suggesting high sensitivity of these selenoproteins on changes in dietary Se intake. Similar results were also reported in rat [13] and pig liver [15]. Furthermore, elevated muscle *SEPN1* mRNA levels by dietary Se supplementation (0.65 mg Se/kg) were consistent with the fact, that the muscle is the major source of this protein [38], and results of previous studies have clearly shown the possible role of SEPN1 protein during muscle development, cell proliferation, and regeneration [38]. The effect of Se concentration on *SEPN1* expression has been also studied in pig liver [15] and chicken muscles [39]. Zhang et al. [39] reported that high-Se diet (1.50 mg Se/kg), compared with a diet containing 0.15 mg Se/kg resulted in the elevation of *SEPN1* mRNA levels in skeletal muscles. In contrast to the muscle, the effect of Se excess on *SEPN1* mRNA level in pig liver appeared to be insignificant [15].

The downregulation of *GPX2*, *SEPM*, and *SEPHS2* mRNA levels in the liver of S lambs fed 0.65 mg Se/kg, compared with C fed 0.15 mg Se/kg, appears to be unique. Because these decreases were not noticed in the muscle, the expression of *GPX2*, *SEPM*, and *SEPHS2* in the liver seems to be more sensitive to the higher level of dietary Se and/or exposure of liver cells to oxidative stress. Zhou et al. [15] reported that *GPX2* mRNA level in pig pituitary (fed 0.3 mg Se/kg) was significantly higher than in the liver and decreased in the pituitary of pig fed 3.0 mg Se/kg. Opposite trend has been observed in chicken liver [39], and rat muscle [13]. In case of *SEPHS2*, pigs supplemented 0.3 and 3.0 mg Se/kg had significantly higher *SEPHS2* mRNA level in the heart compared with those with Se deficiency in the diet [11, 15]. Both *GPX2* and *SEPHS2* are high in the selenoproteins hierarchy and their mRNA expression is little affected, therefore significantly decreased *GPX2*, *SEPM*, and *SEPHS2* in the liver of S group (0.65 mg Se/kg) may be a consequence of higher Se toxicity in the form of selenate. Nevertheless, our data showed that these genes may play protective role by the reduction of oxidative stress toxicity in the liver, but also that tissue-specific regulatory mechanisms controlling their mRNA expression are involved. A recent finding suggest that effect of Se on the mRNA level of these genes is achieved not only through pre-translational mechanisms, but also in the post-transcriptional step by altering the miRNAs profile [40].

It is noteworthy, that results of our study showed lack of common pattern of the *GPX1*, *GPX2*, and *GPX4* expression. Similarly to previous findings reported in rats [41] and pig [15], level of the *GPX4* mRNA in the liver and muscle of both groups of lambs exhibited insignificant response to changes in Se status. Our results suggest that *GPX1* and *GPX2* mRNA may be more sensitive to regulation by Se status than *GPX4*, and different response of mRNA expression to dietary Se might exist between selenoproteins *GPX1*, *GPX2*, and *GPX4*. In the case of *SEPP1*, the high hepatic *SEPP1* mRNA level in comparison with expression of other selenoprotein genes is consistent with the fact, that liver is the major source of plasma SEPP1 and is responsible for Se transport to skeletal muscles and other organs, where Se levels are maintained by means of a ApoER2 receptor-mediated uptake of SEPP1 [42].

### Effect of Se Supplementation on mRNA Level of Lipid Metabolism Genes in the Liver and Muscle

The findings on the Se dietary regulation of lipid metabolism-related genes in ruminants are very limited, and, to our knowledge, the gene expression patterns of *PON1*, *LXRα*, *LPL*, *APOE*, *PPARα*, and *DHCR24* in the liver and muscle of lambs have not been previously reported. Our results showed that changes in Se supply not only lead to changes in the mRNA expression of selenoproteins but also significantly alter mRNA level of the genes, playing essential role in the regulation of the triglycerides and fatty acids metabolism. In our study, we showed that Se supplementation significantly upregulated *LPL* mRNA expression in the liver and muscle of S (0.65 mg Se/kg) group of lambs. The main function of LPL is to hydrolyze triglycerides (TG) in lipoproteins and release free fatty acids, and its activity is critical for TG-rich lipoprotein clearance. Moreover, the LPL activity also affects HDL level in the blood circulation; a decrease of LPL activity leads to decreased HDL concentration. Observed in our study, elevated level of *LPL* mRNA expression in the muscle (*P* < 0.01) of S group of lambs could be associated with higher utilization of fatty acids as energy sources or building blocks for TG, resulting in decrease of the TG in the lamb’s meat. The effect of high-Se diet on *LPL* mRNA expression has been previously reported by Pinto et al. [24] who showed that in pigs, high-Se diet increased mRNA expression of *LPL* in the adipose tissue. Moreover, elevated mRNA levels of these genes were significantly correlated with higher activity of plasma GPX1, MSRB1, SELS, and SEPP1 [20].

In our study, the *APOE* gene was also found to be affected by Se supplementation, and similarly to *LPL*, significantly higher *APOE* mRNA levels were found in the muscle vs. liver, which was consistent with the involvement of tissue-specific mechanisms controlling their expression [43]. Although this protein is synthesized primarily in liver, *APOE* transcripts were found in the kidney [43], adipose tissue [44], and in the skeletal muscles [45]. Earlier studies have clearly demonstrated the role of APOE in many steps of lipoprotein homeostasis such as production, delivery, and utilization of cholesterol in the body [46]. In the plasma, APOE is associated with very low-density lipoproteins (VLDL), chylomicron remnants, and a subset of HDL particles [46]; it is also a high affinity ligand for the LDL receptor [47]. It was reported that a Se-deficient diet or targeted removal of a gene that causes complete loss of selenoproteins expression, results in increased plasma cholesterol concentration along with an increase in APOE levels [22]. It was speculated, that this increase was related to higher HDL fraction, which is rich in APOE. Potential mechanisms that may explain these associations are not clear, but recent studies on knock-out mouse (*Trsp*^−^/*Trsp*^−^) models provided strong evidence, that several selenoproteins such as GPX1, DIO1, SELK, SEPP1, and SEP15 are responsible for the role of Se in the APOE and cholesterol metabolism [22]. The observed common patterns of *LPL* and *APOE* mRNA expression in the LD muscle of S (0.65 mg Se/kg) group of lambs may indicate a bidirectional supportive role of these genes in the delivery and metabolism of TG-rich lipoproteins [48]. It was reported that LPL mediates the uptake of APOE-containing lipoproteins via LDL receptor-related protein [49] and that *APOE* polymorphisms appear to modulate LPL activity [44]. In addition, muscle *LPL* and *APOE* mRNA expression may be more sensitive to regulation by dietary Se status. It is therefore likely that Se may regulate lipid metabolism in cells through *LPL* and *APOE*, which may have selective roles for either cholesterol or fatty acid metabolism.

Interestingly, outcomes of our study showed that Se supplementation did not affected mRNA expression of *PON1*, *LXRα*, *PPARα*, and *DHCR24* genes, but relatively high *PON1* and *LXRα* mRNA expression levels were observed in the liver of both experimental groups of lambs. In agreement with previous report [50], high levels of the *PON1* and *LXRα* transcripts were found in tissues involved in maintaining lipid homeostasis, such as liver, adipocytes, and macrophages [51]. Also, both *PON1* and *LXRα* have a key role in the control of cholesterol homeostasis and fatty acid metabolism [51]. Recently, the beneficial effect of Se supplementation on plasma PON1 activity in the diabetic rats has been investigated [52], but similar to our results, mRNA expression of *PON1* in the liver was not affected in the Se treated groups [53]. These findings suggest that *PON1* expression may possibly be regulated by Se status at post-transcriptional and/or translational levels.

In summary, our study confirmed that, in lambs, similarly to other species, mRNA expression patterns of several selenoproteins, especially *GPX1*, *SEPN1*, *SEP15*, *SEPM*, and *SEPHS2* highly depend on dietary Se levels, but their expression is ruled by hierarchical principles, and they are regulated by tissue-specific mechanisms controlling their expression. Furthermore, the study showed that changes Se intake leads to different levels of gene expression related with lipid metabolism. The results of our study suggests that Se supplementation promote higher levels of the *LPL* and *APOE* gene expression, especially in skeletal muscle and possibly in fatty acid utilization and TG metabolism. Our results provide new insights into the role of Se in these metabolic pathways, indicating that Se is not only an important antioxidant but also a regulator of gene expression. Therefore, further studies on dietary Se levels need be carried out to help us better understand the interactions between nutrients and metabolic pathways.

## Abbreviations

*APOE*: Apolipoprotein *E*
ApoER2: Apolipoprotein E receptor 2
*DHCR24*: 24-dehydrocholesterol reductase
BD: Basal diet
*ACTB*: β-actin
*FOXO1*: Forkhead box protein O1
*GAPDH*: Glyceraldehyde 3-phosphate dehydrogenase gene
DM: Dry matter
*GPX1*: Glutathione peroxidase 1
*GPX2*: Glutathione peroxidase 2
*GPX4*: Glutathione peroxidase 4
HDL: High-density lipoprotein
LDL: Low-density lipoprotein
*LPL*: Lipoprotein lipase
*LXRα*: Liver X receptor α
miRNA: Micro-ribonucleic acid
*MSRB1*: Methionine sulfoxide reductase B1
NRC: National Research Council
*PGC*-*1α*: Peroxisome proliferator-activated receptor-gamma coactivator 1 alpha
*PON1*: Paraoxonase 1
*PPARα*: Peroxisome proliferator-activated receptor *α*
PUFAs: Polyunsaturated fatty acids
CLA: Conjugated linoleic acid
qPCR: Quantitative polymerase chain reaction
*SEPHS2*: Selenophosphate synthetase 2
*SEPM*: Selenoprotein M
*SELO*: Selenoprotein O
*SEPN1*: Selenoprotein N1
*SEPP1*: Selenoprotein 1
*SEP15*: Selenoprotein *15*
*SEPW1*: Selenoprotein W1
*SREPP*-*1*: Sterol regulatory element-binding protein 1
*TXNRD1*: Thioredoxin reductase 1
TG: Triglycerides
UTR: Untranslated region
